# Supplementary material for: The impact of COVID-19 pandemic on fertility behaviour in Indian states: Evidence from the National Family Health Survey (2019/21)
Source: PLoS One. 2024 Dec 6;19(12):e0314800. doi: 10.1371/journal.pone.0314800 (PMC11623806; doi:10.1371/journal.pone.0314800)
Supplement: S1 Checklist — (DOC) [file pone.0314800.s001.doc]

STROBE Statement—Checklist of items that should be included in reports of ***cross-sectional studies***

|  | Item No | Recommendation |
| --- | --- | --- |
| **Title and abstract** | 1 | (*a*) Indicate the study’s design with a commonly used term in the title or the abstract |
|  |  | (*b*) Provide in the abstract an informative and balanced summary of what was done and what was found  **Authors’ response:** Done. Lines 33–354. |
| Introduction | | |
| Background/rationale | 2 | Explain the scientific background and rationale for the investigation being reported  **Authors’ response:** Done. Lines 64–108. |
| Objectives | 3 | State specific objectives, including any prespecified hypotheses. **Authors’ response:** Done. Lines 138–146. |
| Methods | | |
| Study design | 4 | Present key elements of study design early in the paper  **Authors’ response:** Done. Lines 227–229 |
| Setting | 5 | Describe the setting, locations, and relevant dates, including periods of recruitment, exposure, follow-up, and data collection  **Authors’ response:** Done. Lines 224–258. |
| Participants | 6 | (*a*) Give the eligibility criteria, and the sources and methods of selection of participants  **Authors’ response:** Done. Lines 256–284. |
| Variables | 7 | Clearly define all outcomes, exposures, predictors, potential confounders, and effect modifiers. Give diagnostic criteria, if applicable  **Authors’ response:** Done. Lines 286–290 and lines 300–304. |
| Data sources/ measurement | 8* | For each variable of interest, give sources of data and details of methods of assessment (measurement). Describe comparability of assessment methods if there is more than one group  **Authors’ response:** Done. Lines 287­–317. |
| Bias | 9 | Describe any efforts to address potential sources of bias  **Authors’ response:** Done. Lines 258–261, lines 345–346, and lines 353–356. |
| Study size | 10 | Explain how the study size was arrived at  **Authors’ response:** Done. Lines 256–280. |
| Quantitative variables | 11 | Explain how quantitative variables were handled in the analyses. If applicable, describe which groupings were chosen and why  **Authors’ response:** Done. Lines 292–295. |
| Statistical methods | 12 | (*a*) Describe all statistical methods, including those used to control for confounding  **Authors’ response:** Done. Lines 319–346. |
|  |  | (*b*) Describe any methods used to examine subgroups and interactions  **Authors’ response:** Done. Lines 327–342. |
|  |  | (*c*) Explain how missing data were addressed  **Authors’ response:** See Figure 3. |
|  |  | (*d*) If applicable, describe analytical methods taking account of sampling strategy  **Authors’ response:** Done. Lines 319–324. |
|  |  | (*e*) Describe any sensitivity analyses  **Authors’ response: NA** |
| Results | | |
| Participants | 13* | (a) Report numbers of individuals at each stage of study—eg numbers potentially eligible, examined for eligibility, confirmed eligible, included in the study, completing follow-up, and analysed  **Authors’ response:** Done. Lines 253–278. |
|  |  | (b) Give reasons for non-participation at each stage  **Authors’ response:** Done. Lines 253–278. |
|  |  | (c) Consider use of a flow diagram  **Authors’ response:** Done. Fig 3. |
| Descriptive data | 14* | (a) Give characteristics of study participants (eg demographic, clinical, social) and information on exposures and potential confounders  **Authors’ response:** Done. Lines 353–365. |
|  |  | (b) Indicate number of participants with missing data for each variable of interest  **Authors’ response:** Done. Fig 3. |
| Outcome data | 15* | Report numbers of outcome events or summary measures  **Authors’ response:** Done. Lines 375­–398. |
| Main results | 16 | (*a*) Give unadjusted estimates and, if applicable, confounder-adjusted estimates and their precision (eg, 95% confidence interval). Make clear which confounders were adjusted for and why they were included  **Authors’ response:** Done. Lines 399–413, and S3, S4, and S5 Tables in supplementary table file named “S1, S2, S3, S4, and S5 Tables” |
|  |  | (*b*) Report category boundaries when continuous variables were categorized  **Authors’ response:** Done. Please see the variables in Tables 2­–4. |
|  |  | (*c*) If relevant, consider translating estimates of relative risk into absolute risk for a meaningful time period  **Authors’ response:** Not Applicable. |
| Other analyses | 17 | Report other analyses done—eg analyses of subgroups and interactions, and sensitivity analyses  **Authors’ response:** Not Applicable. |
| Discussion | | |
| Key results | 18 | Summarise key results with reference to study objectives  **Authors’ response:** Done. Lines 459–468. |
| Limitations | 19 | Discuss limitations of the study, taking into account sources of potential bias or imprecision. Discuss both direction and magnitude of any potential bias  **Authors’ response:** Done. Lines 564–585. |
| Interpretation | 20 | Give a cautious overall interpretation of results considering objectives, limitations, multiplicity of analyses, results from similar studies, and other relevant evidence  **Authors’ response:** Done. Lines 469–556. |
| Generalisability | 21 | Discuss the generalisability (external validity) of the study results  **Authors’ response:** Done. Lines 586–613. |
| Other information | | |
| Funding | 22 | Give the source of funding and the role of the funders for the present study and, if applicable, for the original study on which the present article is based  **Authors’ response:** Information has been provided in the journal submission system. |

*Give information separately for exposed and unexposed groups.

**Note:** An Explanation and Elaboration article discusses each checklist item and gives methodological background and published examples of transparent reporting. The STROBE checklist is best used in conjunction with this article (freely available on the Web sites of PLoS Medicine at http://www.plosmedicine.org/, Annals of Internal Medicine at http://www.annals.org/, and Epidemiology at http://www.epidem.com/). Information on the STROBE Initiative is available at www.strobe-statement.org.
