# Supplementary material for: The impact of COVID-19 pandemic on fertility behaviour in Indian states: Evidence from the National Family Health Survey (2019/21)
Source: PLoS One. 2024 Dec 6;19(12):e0314800. doi: 10.1371/journal.pone.0314800 (PMC11623806; doi:10.1371/journal.pone.0314800)
Supplement: S1 Table — (DOCX) [file pone.0314800.s003.docx]

**S1 Table. Selected weighted cases for each dependent variable interviewed in pre- and post-lockdown periods in the selected Indian states and UTs, 2019/21.**

| **State/union territory** | **Next birth within 2 years**  **Number (%)** | | **Contraceptive use**  **Number (%)** | | **Had sex in last 30 days**  **Number (%)** | |
| --- | --- | --- | --- | --- | --- | --- |
|  | **Pre-lockdown** | **Post-lockdown** | **Pre-lockdown** | **Post-lockdown** | **Pre-lockdown** | **Post-lockdown** |
| Pooled data | 24,375 (43.1) | 32,186 (56.9) | 108,684 (43.3) | 142,125 (56.7) | 22,082 (41.5) | 31,129 (58.5) |
| Punjab | 734 (27.7) | 1,1916 (72.3) | 5,392 (35.1) | 9,957 (64.9) | 1,183 (33.7) | 2,325 (66.3) |
| Uttarakhand | 795 (44.6) | 988 (55.4) | 4,004 (43.8) | 5,147 (56.2) | 794 (45.0) | 970 (55.0) |
| Haryana | 730 (25.0) | 2,195 (75.0) | 4,125 (26.2) | 11,620 (73.8) | 736 (22.9) | 2,472 (77.1) |
| Delhi | 921 (60.4) | 604 (39.6) | 4,556 (61.3) | 2,881 (38.7) | 940 (55.1) | 765 (44.9) |
| Rajasthan | 5,838 (77.0) | 1,741 (23.0) | 23,926 (77.7) | 6,859 (22.3) | 4,988 (76.3) | 1,553 (23.7) |
| Uttar Pradesh | 5,698 (37.8) | 9,390 (62.2) | 22,850 (36.4) | 39,850 (63.6) | 4,676 (34.3) | 8975 (65.7) |
| Arunachal Pradesh | 1,464 (48.1) | 1,577 (51.9) | 6,363 (46.6) | 7,295 (53.4) | 1,260 (43.0) | 1,673 (57) |
| Jharkhand | 1,874 (38.9) | 2,939 (61.1) | 7,860 (40.3) | 11,623 (59.7) | 1,486 (36.7) | 2,562 (63.3) |
| Odisha | 1,780 (38.7) | 2,825 (61.3) | 9,100 (45.1) | 11,083 (54.9) | 1,819 (41.9) | 2,521 (58.1) |
| Chhattisgarh | 1,972 (43.1) | 2,601 (56.9) | 8,390 (44.3) | 10,537 (55.7) | 1,949 (45.7) | 2,316 (54.3) |
| Madhya Pradesh | 2,964 (40.9) | 4,277 (59.1) | 14,363 (41.0) | 20,640 (59.0) | 2,729 (40.1) | 4,074 (59.9) |
| Tamil Nadu | 1,167 (30.4) | 2,671 (69.6) | 5,947 (32.2) | 12,528 (67.8) | 1,171 (30.9) | 2,623 (69.1) |
| Puducherry | 392 (84.7) | 71 (15.3) | 2,105 (86.4) | 331 (13.6) | 470 (80.8) | 112 (19.2) |

**Data source:** Authors’ calculation using NFHS-5, 2019/21.

**Note:** Summations of the weighted numbers for the states do not match the pooled numbers because the states’ numbers were weighted by the state-level weights and the pooled numbers were weighted by the country-level weights. Summations of the pre-lockdown and post-lockdown cases may not match the total number because of rounding up of the values.
