# Supplementary material for: The impact of COVID-19 pandemic on fertility behaviour in Indian states: Evidence from the National Family Health Survey (2019/21)
Source: PLoS One. 2024 Dec 6;19(12):e0314800. doi: 10.1371/journal.pone.0314800 (PMC11623806; doi:10.1371/journal.pone.0314800)
Supplement: S2 Table — (DOCX) [file pone.0314800.s004.docx]

**S2 Table. Percentage of the pooled women selected for each analysis by interview period and selected characteristics, India 2019/21.**

| **Characteristics** | **Desire for a birth within 2 years**  **% (weighted number)** | | **Contraceptive use**  **% (weighted number)** | | **Had sex in the last 30 days**  **% (weighted number)** | |
| --- | --- | --- | --- | --- | --- | --- |
|  | **Pre-lockdown** | **Post-lockdown** | **Pre-lockdown** | **Post-lockdown** | **Pre-lockdown** | **Post-lockdown** |
| Current age  15–24  25–34  35–49 | 50.4 (12,292)  42.8 (10,432)  6.8 (1,651) | 45.8 (14,726)  46.9 (15,088)  7.4 (2,372) | 16.8 (18,208)  38.4 (41,755)  44.8 (48,721) | 15.0 (21,369)  38.6 (54,880)  46.4 (65,876) | 35.8 (7,897)  29.6 (6,538)  34.6 (7,648) | 34.6 (10,761)  30.5 (9,498)  34.9 (10,870) |
| Number of children  0  1–2  >2 | 35.4 (8,633)  59.4 (14,481)  5.2 (1,261) | 34.7 (11,157)  60.5 (19,470)  4.8 (1,560) | 9.5 (10,341)  52.8 (57,333)  37.7 (41,011) | 9,5 (13,492)  54.2 (77,092)  36.3 (51,542) | 32.3 (7,142)  39.2 (8,666)  28.4 (6,275) | 33.3 (10,375)  40.4 (12,578)  26.3 (8,176) |
| Religion  Hindu  Muslim  Christian  Other | 87.5 (21,316)  9.2 (2,253)  0.7 (175)  2.6 (630) | 83.4 (26,834)  11.3 (3,628)  1.9 (618)  3.4 (1,107) | 88.0 (95,593)  7.7 (8,335)  0.7 (808)  3.6 (3,948) | 84.6 (120,234)  9.8 (13,890)  1.7 (2,469)  3.9 (5,532) | 87.9 (19,407)  7.8 (1,712)  0.7 (149)  3.7 (814) | 83.5 (26,004)  10.3 (3,211)  1.8 (562)  4.3 (1,353) |
| Caste  Scheduled caste/tribe  Other backward classes  Other  Don’t know/missing | 33.2 (8,104)  47.0 (11,465)  17.9 (4,359)  1.8 (446) | 35.6 (11,458)  47.9 (15,415)  16.5 (4,948)  1.4 (365) | 31.5 (34,288)  46.9 (50,978)  19.5 (21,191)  2.0 (2,228) | 33.6 (47,802)  48.3 (68,659)  16.8 (23,863)  1.3 (1,802) | 32.5 (7,175)  46.3 (10,225)  19.3 (4,260)  1.9 (423) | 34.0 (10,582)  48.0 (14,946)  16.7 (5,195)  1.3 (407) |
| Level of schooling  No schooling  <7 years complete  7–9 years complete  10–11 years complete  12^+^ years complete | 17.2 (4,204)  12.4 (3,031)  22.9 (5,580)  13.0 (3,178)  34.4 (8,382) | 13.9 (4,465)  10.9 (3,518)  21.5 (6,911)  12.8 (4,124)  40.9 (13,168) | 32.4 (35,227)  16.7 (18,197)  20.4 (22,204)  9.8 (10,678)  20.6 (22,379) | 28.6 (40685)  15.5 (21,965)  19.9 (28,327)  10.8 (15,389)  25.2 (35,760) | 26.0 (5,749)  14.6 (3,217)  22.8 (5,024)  12.8 (2,819)  23.9 (5,273) | 22.0 (6,843)  13.2 (4,096)  21.5 (6,707)  14.1 (4,399)  29.2 (9,084) |
| Wealth status  Poorest  Poorer  Middle  Richer  Richest | 17.4 (4,239)  20.6 (5,029)  20.4 (4,972)  19.9 (4,848)  21.7 (5,286) | 19.2 (6,189)  18.4 (5,936)  18.3 (5,904)  20.0 (6,430)  24.0 (7,727) | 18.1 (19,685)  20.8 (22,582)  20.1 (21,807)  19.4 (21,057)  21.7 (23,553) | 19.4 (27,529)  18.7 (26,519)  18.7 (26,524)  19.6 (27,920)  23.7 (33,632) | 17.8 (3,932)  22.1 (4,879)  20.2 (4,451)  19.4 (4,284)  20.5 (4,537) | 19.3 (6,023)  19.1 (5,942)  19.2 (5,983)  19.2 (5,971)  23.2 (7,210) |
| Residence  Urban  Rural | 28.1 (6,846)  71.9 (17,528) | 29.3 (9,440)  70.7 (22,747) | 29.1 (31,667)  70.9 (77,017) | 30.5 (43,338)  69.5 (98,787) | 29.3 (6,460)  70.7 (15,622) | 30.8 (9,594)  69.2 (21,535) |
| Had media access  Yes  No | 55.7 (13,587)  44.3 (10,787) | 55.2 (17,758)  44.8 (14,429) | 52,9 (57,538)  47.1 (51,146) | 53.4 (75,832)  46.6 (66,293) | 54.7 (12,077)  45.3 (10,006) | 54.6 (16,994)  45.4 (14,135) |
| Total | 100 (24,375) | 100 (32,186) | 100 (108,684) | 100 (142,125) | 100 (22,082) | 100 (31,129) |

**Source:** Authors’ calculation using NFHS-5, 2019/21.

**Note:** The estimates were obtained by applying national level weights. Summations of the weighted numbers and percentages may not match the totals because of rounding up.
