# Supplementary material for: The impact of COVID-19 pandemic on fertility behaviour in Indian states: Evidence from the National Family Health Survey (2019/21)
Source: PLoS One. 2024 Dec 6;19(12):e0314800. doi: 10.1371/journal.pone.0314800 (PMC11623806; doi:10.1371/journal.pone.0314800)
Supplement: S4 Table — (DOCX) [file pone.0314800.s006.docx]

**S4 Table. 95% confidence intervals (CI) and P**-**values of the adjusted odds ratios of using contraception among the women interviewed in pre- and post-lockdown periods in the selected Indian states, 2019/21.**

| **Characteristics** | **Pooled data**  N=250,810 | | **Punjab**  n=15,349 | | **Uttarakhand**  n=9,151 | | **Haryana**  n=15,745 | | **Delhi**  n=7,437 | | **Rajasthan**  n=30,785 | | **Uttar Pradesh**  n=62,700 | | **Arunachal Pradesh**  n=13,658 | | **Jharkhand**  n=19,483 | | **Odisha**  n=20,183 | | **Chhattisgarh**  n=18,927 | | **Madhya Pradesh**  n=35,003 | | **Tamil Nadu**  n=18,475 | | **Puducherry**  n=2,436 | |
| --- | --- | --- | --- | --- | --- | --- | --- | --- | --- | --- | --- | --- | --- | --- | --- | --- | --- | --- | --- | --- | --- | --- | --- | --- | --- | --- | --- | --- |
|  | 95% CI | P | 95% CI | P | 95% CI | P | 95% CI | P | 95% CI | P | 95% CI | P | 95% CI | P | 95% CI | P | 95% CI | P | 95% CI | P | 95% CI | P | 95% CI | P | 95% CI | P | 95% CI | P |
| Interviewed in  Pre-lockdown ®  Post-lockdown | -  0.89–0.95 | -  0.000 | -  1.28–1.74 | -  0.000 | -  0.94–1.28 | -  0.224 | -  0.88–1.13 | -  0.950 | -  1.59–2.31 | -  0.000 | -  0.89–1.12 | -  0.995 | -  0.85–0.95 | -  0.000 | -  1.51–2.12 | -  0.000 | -  0.62–0.78 | -  0.000 | -  0.75–0.95 | -  0.004 | -  0.76–1.02 | -  0.094 | -  0.98–1.20 | -  0.105 | -  1.01–1.29 | -  0.030 | -  0.99–2.45 | -  0.056 |
| Current age  15–24 ®  25–34  35–49 | -  1.79–1.92  2.23–2.42 | -  0.000  0.000 | -  1.44–2.06  1.51–2.19 | -  0.000  0.000 | -  1.53–2.32  1.87–3.05 | -  0.000  0.000 | -  2.16–2.85  2.80–3.82 | -  0.000  0.000 | -  1.31–2.08  1.18–1.94 | -  0.000  0.001 | -  1.73–2.10  3.28–4.10 | -  0.000  0.000 | -  1.47–1.66  1.36–1.58 | -  0.000  0.000 | -  1.06–1.50  1.12–1.61 | -  0.009  0.001 | -  1.83–2.29  2.22–2.82 | -  0.000  0.000 | -  1.53–1.99  1.31–1.74 | -  0.000  0.000 | -  1.74–2.37  3.11–4.41 | -  0.000  0.000 | -  2.38–2.88  3.88–4.89 | -  0.000  0.000 | -  1.68–2.33  2.90–3.99 | -  0.000  0.000 | -  0.96–3.68  1.99–8.21 | -  0.068  0.000 |
| Number of children  0 ®  1–2  >2 | -  9.87–10.90  15.55–17.38 | -  0.000  0.000 | -  12.40–18.69  17.62–28.05 | -  0.000  0.000 | -  6.91–12.15  9.16–17.60 | -  0.000  0.000 | -  13.82–20.54  21.21–34.46 | -  0.000  0.000 | -  8.85–14.45  14.19–25.26 | -  0.000  0.000 | -  6.41–8.35  10.98–14.96 | -  0.000  0.000 | -  5.60–6.64  9.10–10.97 | -  0.000  0.000 | -  2.59–3.81  3.66–5.67 | -  0.000  0.000 | -  9.02–13.71  16.08–25.05 | -  0.000  0.000 | -  11.63–15.99  16.09–23.16 | -  0.000  0.000 | -  21.17–34.44  44.87–75.90 | -  0.000  0.000 | -  11.09–14.97  17.44–24.28 | -  0.000  0.000 | -  73.91–157.10  198.79–433.42 | -  0.000  0.000 | -  15.18–117.60  48.98–435.25 | -  0.000  0.000 |
| Religion  Hindu ®  Muslim  Christian  Other | -  0.56–0.62  0.78–1.00  0.86–0.98 | -  0.000  0.043  0.015 | -  0.29–0.77  0.60–1.36  0.93–1.21 | -  0.003  0.631  0.370 | -  0.51–0.92  -  0.77–2.48 | -  0.011  -  0.277 | -  0.28–0.44  0.09–1.29  0.75–1.20 | -  0.000  0.114  0.637 | -  0.48–0.82  1.59–14.11  0.68–1.53 | -  0.001  0.005  0.938 | -  0.56–0.80  0.34–2.03  0.80–1.51 | -  0.000  0.684  0.551 | -  0.60–0.70  0.11–1.06  0.84–2.02 | -  0.000  0.062  0.238 | -  0.47–1.42  0.84–1.17  0.78–1.10 | -  0.476  0.917  0.403 | -  0.37–0.51  0.69–1.07  0.86–1.23 | -  0.000  0.180  0.770 | -  0.57–1.38  0.68–1.20  0.90–4.22 | -  0.592  0.479  0.089 | -  0.57–1.16  0.51–1.08  0.57–1.85 | -  0.265  0.116  0.920 | -  0.52–0.81  0.54–2.81  0.76–1.76 | -  0.000  0.614  0.509 | -  0.49–0.81  0.82–1.29  0.65–33.38 | -  0.000  0.808  0.127 | -  0.15–0.94  0.27–1.19  0.12–10.31 | -  0.036  0.134  0.918 |
| Caste  Scheduled caste/tribe ®  Other backward classes  Other  Don’t know/missing | -  0.98–1.05  1.06–1.15  0.93–1.13 | -  0.325  0.000  0.568 | -  0.76–1.02  1.03–1.31  0.63–1.18 | -  0.100  0.015  0.357 | -  0.68–1.08  0.80–1.16  0.50–1.07 | -  0.187  0.722  0.104 | -  0.96–1.25  1.05–1.35  0.82–1.84 | -  0.176  0.008  0.312 | -  0.81–1.29  0.85–1.23  0.63–1.11 | -  0.832  0.831  0.222 | -  0.96–1.14  0.97–1.24  0.73–1.35 | -  0.311  0.131  0.958 | -  0.96–1.10  1.04–1.20  0.80–1.53 | -  0.509  0.002  0.547 | -  0.62–1.14  1.25–2.12  0.79–1.31 | -  0.260  0.000  0.890 | -  1.16–1.42  1.01–1.41  0.90–1.71 | -  0.000  0.043  0.193 | -  0.81–1.01  0.77–1.05  0.48–1.08 | -  0.064  0.192  0.112 | -  1.17–1.47  1.07–1.82  1.19–2.12 | -  0.000  0.013  0.002 | -  0.96–1.15  0.81–1.04  0.66–1.05 | -  0.250  0.188  0.113 | -  0.95–1.19  0.54–1.41  0.79–2.86 | -  0.283  0.579  0.213 | -  0.57–1.37  0.38–2.17  0.15–2.58 | -  0.587  0.836  0.500 |
| Level of schooling  No schooling ®  <7 years complete  7–9 years complete  10–11 years complete  12^+^ years complete | -  1.09–1.18  1.02–1.10  0.96–1.05  0.86–0.95 | -  0.000  0.002  0.928  0.000 | -  0.79–1.13  0.81–1.15  0.77–1.12  0.74–1.08 | -  0.549  0.677  0.418  0.245 | -  1.15–1.90  1.06–1.74  0.87–1.61  1.00–1.68 | -  0.002  0.016  0.294  0.052 | -  1.09–1.50  0.90–1.25  0.82–1.18  0.81–1.14 | -  0.003  0.484  0.837  0.641 | -  0.86–1.40  0.89–1.46  0.85–1.42  1.02–1.58 | -  0.442  0.296  0.456  0.036 | -  0.98–1.18  0.81–1.01  0.74–0.99  0.85–1.11 | -  0.146  0.075  0.036  0.635 | -  0.96–1.11  0.99–1.13  0.96–1.15  0.98–1.13 | -  0.371  0.095  0.248  0.169 | -  1.28–1.78  1.22–1.62  1.03–1.45  0.94–1.34 | -  0.000  0.000  0.024  0.196 | -  1.04–1.33  1.04–1.29  0.88–1.15  0.86–1.15 | -  0.011  0.010  0.915  0.935 | -  0.99–1.27  1.04–1.39  1.02–1.43  0.94–1.40 | -  0.080  0.013  0.030  0.184 | -  1.02–1.38  1.04–1.38  0.97–1.48  0.80–1.12 | -  0.028  0.015  0.086  0.535 | -  0.92–1.11  0.77–0.93  0.69–0.92  0.64–0.84 | -  0.814  0.000  0.003  0.000 | -  0.93–1.42  0.93–1.43  0.81–1.29  0.53–0.83 | -  0.206  0.195  0.856  0.000 | -  0.35–2.81  0.24–2.35  0.23–2.57  0.15–1.03 | -  0.994  0.630  0.678  0.056 |
| Wealth status  Poorest ®  Poorer  Middle  Richer  Richest | -  1.10–1.18  1.15–1.25  1.17–1.28  1.19–1.33 | -  0.000  0.000  0.000  0.000 | -  0.89–2.29  0.88–2.12  0.70–1.72  0.68–1.64 | -  0.143  0.163  0.675  0.798 | -  0.85–1.52  0.66–1.15  0.58–1.04  0.58–1.12 | -  0.376  0.342  0.088  0.204 | -  0.90–1.76  1.16–2.19  1.21–2.28  1.25–2.37 | -  0.171  0.004  0.002  0.001 | -  0.14–8.29  0.15–7.48  0.17–8.49  0.17–8.51 | -  0.946  0.945  0.855  0.851 | -  0.94–1.20  0.87–1.11  0.92–1.22  0.90–1.24 | -  0.344  0.765  0.387  0.488 | -  1.05–1.18  1.09–1.25  1.12–1.31  1.24–1.50 | -  0.001  0.000  0.000  0.000 | -  0.85–1.16  0.82–1.16  0.67–1.02  0.77–1.13 | -  0.930  0.776  0.082  0.770 | -  1.26–1.55  1.25–1.66  1.38–1.95  1.10–1.76 | -  0.000  0.000  0.000  0.006 | -  0.89–1.13  0.89–1.19  0.90–1.31  0.85–1.38 | -  0.957  0.717  0.367  0.504 | -  1.07–1.39  1.27–1.76  1.33–1.94  1.35–2.16 | -  0.003  0.000  0.000  0.000 | -  1.00–1.20  1.04–1.30  0.96–1.24  1.09–1.53 | -  0.045  0.009  0.182  0.003 | -  0.70–1.21  0.76–1.30  0.75–1.33  0.61–1.41 | -  0.553  0.963  0.969  0.257 | -  0.13–1.48  0.14–1.00  0.12–1.01  0.12–0.99 | -  0.186  0.051  0.052  0.048 |
| Residence  Urban ®  Rural | -  0.86–0.94 | -  0.000 | -  0.74–1.00 | -  0.049 | .-  0.63–0.93 | -  0.008 | -  0.99–1.30 | -  0.072 | -  0.57–1.38 | -  0.600 | -  0.77–1.06 | -  0.214 | -  0.71–0.84 | -  0.000 | -  0.86–1.35 | -  0.533 | -  0.83–1.14 | -  0.704 | -  0.71–1.03 | -  0.106 | -  0.90–1.33 | -  0.372 | -  1.01–1.31 | -  0.030 | -  0.86–1.10 | -  0.655 | -  0.62–1.67 | -  0.952 |
| Had media access  Yes ®  No | -  0.79–0.84 | -  0.000 | -  0.67–0.84 | -  0.000 | -  0.69–0.96 | -  0.016 | -  0.69–0.87 | -  0.000 | -  0.66–0.91 | -  0.002 | -  0.79–0.93 | -  0.000 | -  0.82–0.91 | -  0.000 | -  0.65–0.82 | -  0.000 | -  0.87–1.10 | -  0.738 | -  0.80–0.98 | -  0.019 | -  0.68–0.87 | -  0.000 | -  0.70–0.82 | -  0.000 | -  0.87–1.15 | -  0.997 | -  0.43–1.38 | -  0.372 |
| Constant | 0.13–0.15 | 0.000 | 0.05–0.15 | 0.000 | 0.13–0.32 | 0.000 | 0.03–0.07 | 0.000 | 0.02–1.14 | 0.068 | 0.18–0.29 | 0.000 | 0.21–0.28 | 0.000 | 0.18–0.38 | 0.000 | 0.05–0.10 | 0.000 | 0.18–0.31 | 0.000 | 0.02–0.04 | 0.000 | 0.07–0.12 | 0.000 | 0.01–0.02 | 0.000 | 0.02–0.68 | 0.018 |
| **Model fit statistics** | | | | | | | | | | | | | | | | | | | | | | | | | | | | |
| F-statistic | - | 0.0000 | - | 0.0000 | - | 0.0000 | - | 0.0000 | - | 0.0000 | - | 0.0000 | - | 0.0000 | - | 0.0000 | - | 0.0000 | - | 0.0000 | - | 0.0000 | - | 0.0000 | - | 0.0000 | - | 0.0000 |
| ICC | 0.132–0.144 | - | 0.129–0.181 | - | 0.039–0.091 | - | 0.071–0.112 | - | 0.066–0.147 | - | 0.146–0.190 | - | 0.100–0.120 | - | 0.158–0.226 | - | 0.065–0.098 | - | 0.101–0.141 | - | 0.150–0.204 | - | 0.156–0.194 | - | 0.073–0.113 | - | 0.039–0.149 | - |

Source: Authors’ calculation using NFHS-5, 2019/21.

Note: CI = Confidence interval. ICC = Intraclass correlation coefficient. The number of cases in pooled data (N) and in states’ data (n) are weighted with, respectively, national level and state level weights. Confidence intervals were produced using the delta method.
