# Supplementary material for: The impact of COVID-19 pandemic on fertility behaviour in Indian states: Evidence from the National Family Health Survey (2019/21)
Source: PLoS One. 2024 Dec 6;19(12):e0314800. doi: 10.1371/journal.pone.0314800 (PMC11623806; doi:10.1371/journal.pone.0314800)
Supplement: Attachment — Submitted filename: Response to Reviewers.docx [file pone.0314800.s008.docx]

**Point-wise response to editor’s and reviewers’ comments**

**Note:** All the line numbers refer to the file named “Revised Manuscript with Track Changes”.

Journal requirements:

When submitting your revision, we need you to address these additional requirements.

1. Please ensure that your manuscript meets PLOS ONE's style requirements, including those for file naming. The PLOS ONE style templates can be found at

<https://journals.plos.org/plosone/s/file?id=wjVg/PLOSOne_formatting_sample_main_body.pdf> and

<https://journals.plos.org/plosone/s/file?id=ba62/PLOSOne_formatting_sample_title_authors_affiliations.pdf>

# Authors’ Response

## Formatted as per instructions.

2. For studies involving third-party data, we encourage authors to share any data specific to their analyses that they can legally distribute. PLOS recognizes, however, that authors may be using third-party data they do not have the rights to share. When third-party data cannot be publicly shared, authors must provide all information necessary for interested researchers to apply to gain access to the data. (<https://journals.plos.org/plosone/s/data-availability#loc-acceptable-data-access-restrictions>)

For any third-party data that the authors cannot legally distribute, they should include the following information in their Data Availability Statement upon submission:

a) A description of the data set and the third-party source

b) If applicable, verification of permission to use the data set

c) Confirmation of whether the authors received any special privileges in accessing the data that other researchers would not have

d) All necessary contact information others would need to apply to gain access to the data

# Authors’ Response

All the line numbers refer to the file named “Revised Manuscript with Track Changes”. We have added notes on the availability of data in the data subsection under the methodology section in the manuscript and manuscript submission process. For seeing the notes in the manuscript, please see the lines 208–210.

3. We note that Figures 2 and 4 in your submission contain [map/satellite] images which may be copyrighted. All PLOS content is published under the Creative Commons Attribution License (CC BY 4.0), which means that the manuscript, images, and Supporting Information files will be freely available online, and any third party is permitted to access, download, copy, distribute, and use these materials in any way, even commercially, with proper attribution. For these reasons, we cannot publish previously copyrighted maps or satellite images created using proprietary data, such as Google software (Google Maps, Street View, and Earth). For more information, see our copyright guidelines: <http://journals.plos.org/plosone/s/licenses-and-copyright>.

We require you to either (1) present written permission from the copyright holder to publish these figures specifically under the CC BY 4.0 license, or (2) remove the figures from your submission:

a. You may seek permission from the original copyright holder of Figures 2 and 4 to publish the content specifically under the CC BY 4.0 license.

We recommend that you contact the original copyright holder with the Content Permission Form (<http://journals.plos.org/plosone/s/file?id=7c09/content-permission-form.pdf>) and the following text:

“I request permission for the open-access journal PLOS ONE to publish XXX under the Creative Commons Attribution License (CCAL) CC BY 4.0 (<http://creativecommons.org/licenses/by/4.0/>). Please be aware that this license allows unrestricted use and distribution, even commercially, by third parties. Please reply and provide explicit written permission to publish XXX under a CC BY license and complete the attached form.”

Please upload the completed Content Permission Form or other proof of granted permissions as an "Other" file with your submission.

In the figure caption of the copyrighted figure, please include the following text: “Reprinted from [ref] under a CC BY license, with permission from [name of publisher], original copyright [original copyright year].”

b. If you are unable to obtain permission from the original copyright holder to publish these figures under the CC BY 4.0 license or if the copyright holder’s requirements are incompatible with the CC BY 4.0 license, please either i) remove the figure or ii) supply a replacement figure that complies with the CC BY 4.0 license. Please check copyright information on all replacement figures and update the figure caption with source information. If applicable, please specify in the figure caption text when a figure is similar but not identical to the original image and is therefore for illustrative purposes only.

The following resources for replacing copyrighted map figures may be helpful:

USGS National Map Viewer (public domain): <http://viewer.nationalmap.gov/viewer/>

The Gateway to Astronaut Photography of Earth (public domain): <http://eol.jsc.nasa.gov/sseop/clickmap/>

Maps at the CIA (public domain): <https://www.cia.gov/library/publications/the-world-factbook/index.html> and <https://www.cia.gov/library/publications/cia-maps-publications/index.html>

NASA Earth Observatory (public domain): <http://earthobservatory.nasa.gov/>

Landsat: <http://landsat.visibleearth.nasa.gov/>

USGS EROS (Earth Resources Observatory and Science (EROS) Center) (public domain): [http://eros.usgs.gov/#](http://eros.usgs.gov/)

Natural Earth (public domain): <http://www.naturalearthdata.com/>

# Authors’ Response

## The maps (Figures 2 & 4) used in our article were not copyrighted. We generated Fig 2 using the shape file from the database of Runfold et al. (Runfola D, Anderson A, Baier H, Crittenden M, Dowker E, Fuhrig S, et al. geoBoundaries: A global database of political administrative boundaries. PLoS ONE. 2020; 15(4):e0231866.). We have clarified the note with the title of the Fig 2 (please see the title and note of Fig 2). The Fig 4 was produced using the shape file provided by the 2019/21 National Family and Health Survey (available on <https://dhsprogram.com/> subject to an online request) and the database of Runfold et al. (we have clarified these through the notes in Fig 4). Both figures were produced using ArcGIS desktop version. We have explained these in lines 309–315. The <https://dhsprogram.com/> granted us access to the shape file with due process, and the database of Runfold et al. is freely available to everyone. Therefore, we don’t need to obtain any permission for using those maps, and there is no restriction in using and distributing the figures.

4. Please review your reference list to ensure that it is complete and correct. If you have cited papers that have been retracted, please include the rationale for doing so in the manuscript text, or remove these references and replace them with relevant current references. Any changes to the reference list should be mentioned in the rebuttal letter that accompanies your revised manuscript. If you need to cite a retracted article, indicate the article’s retracted status in the References list and also include a citation and full reference for the retraction notice.

# Authors’ Response

We have newly added following references:

41. Murphy KR, Myors B. Statistical power analysis: A simple and general model for traditional and modern hypothesis tests. Mahwah, NJ: Lawrence Erlbaum [Internet]. 1998 [cited 2024 Aug 17]. Available from: <https://www.routledge.com/Statistical-Power-Analysis-A-Simple-and-General-Model-for-Traditional-and-Modern-Hypothesis-Tests-Fifth-Edition/Myors-Murphy/p/book/9781032283005>

42. LeBreton JM, Senter JL. Answers to 20 questions about interrater reliability and interrater agreement. Organ Res Methods. 2008; 11(4):815–52.

43. StataCorp. Stata: Release 16. Statistical Software. College Station, TX: StataCorp LLC [Internet]. 2019 [cited 2020 Apr 20]. Available from: https://www.stata.com/manuals/me.pdf

Besides these we have checked the correctness and completeness of the references.

# Authors’ overall Response

## The authors are thankful to the editor and reviewers for reviewing this article and providing constructive suggestions for improving our article. We have effected all the changes suggested by the editor and reviewers with due explanation. Besides the suggestions of the reviewers, we tried to improve our article by clarifying sentences and correcting typographic mistakes. A list of such corrections are as follows (All the line numbers refer to the file named “Revised Manuscript with Track Changes”.):

i) We have included the word pandemic in the title to make that more appropriate. Please see the line 1
ii) In Fig 3, in a box we wrote “Case selection for analyzing modern contraceptive use”, but that should be “Case selection for analysing contraceptive use”, and we have corrected accordingly.
iii) We have revised following lines to improve the sentences: l27–l28, l35, l36, l42, l43, l44, l49–l57, l349, l375–l377, l389–l391, l403–l405, l510–512, l522–524, l556, l561
iv) We have corrected some typographic mistakes in the tables as well, which are as follows: subcategories under current age and caste in Table 2, 3 and 4, Odds ratios for religion in Tamil Nadu and Puducherry in Table 2, Odds ratios for wealth status in Delhi in Table 2, Odds ratio for level of schooling in Puducherry in Table 4, Odds ratio for wealth status in Delhi and Puducherry in Table 4, replacing modern contraceptive use with contraceptive use in S1 Table, and including data for number of children and had media access in S2 Table,
v) We have corrected typographic mistakes in lines l250, l257, l262, l284, l288, l289, l324–l328.

**Additional Editor Comments:**

1. Provide a structured abstract.

# Authors’ Response

## We have made a structured abstract.

2. The 95% CI of odds ratios and exact P values are not mentioned in Tables 2, 3 and 4 of the adjusted odds ratios. Please, organize the tables properly.

# Authors’ Response

## Thanks for this suggestion, because CIs and P values are very important. We have provided CIs and P values in supplementary tables because of the following reasons. We have fitted 14 models for each dependent variable; as a result, our tables containing the results of multivariate analysis (Table 2, 3, and 4) already have become very wide. If we put CI and P value in addition to odds ratio in the same table, each of the table will become extremely wide, which will reduce the readability of the tables and paper. Considering all these facts, we have put the CIs and P values for Table 2, 3, and 4 in the supplementary S3, S4, and S5 Tables, respectively. You can find those supplementary tables in supplementary file “S1, S2, S3, S4, and S5 Tables”.

3. The mixed logit model was used in the study. Adjusted odds ratios are mentioned. However, other statistical details of the model for the pooled data are lacking in the manuscript, e.g., the goodness of fit test, Bayesian information criterion etc.

# Authors’ Response

## In revision, we have reanalyzed our data using Stata 17.0 (the initial analyses were done with the Stata 15.0). The Stata 17.0 can produce intraclass correlation coefficients (ICC). We have put ICC and result of F-test for each model (Please see the last two rows of the Tables 2, 3, and 4). F-statistic and ICC could be considered goodness of fit. We have explained about F-statistic and ICC in the statistical analysis section (please see the lines 297–302). We have also explained these in the result section in lines 407–421. We employed mixed-effects logit regression analysis with complex sampling design, which did not produce Bayesian Information Criterion (BIC) and Akaike Information Criterion (AIC). BIC and AIC are usually used for model comparison. In our study, we employed logit model because our variables were categorical. As we did not fit any model other than logit, we did not have any model to compare with using BIC or AIC.

## [Note: HTML markup is below. Please do not edit.]

## **Reviewers' comments:** Reviewer's Responses to Questions

**Comments to the Author**

1. Is the manuscript technically sound, and do the data support the conclusions?

The manuscript must describe a technically sound piece of scientific research with data that supports the conclusions. Experiments must have been conducted rigorously, with appropriate controls, replication, and sample sizes. The conclusions must be drawn appropriately based on the data presented.

Reviewer #1: Yes

Reviewer #2: Yes

2. Has the statistical analysis been performed appropriately and rigorously?

Reviewer #1: Yes

Reviewer #2: Yes

3. Have the authors made all data underlying the findings in their manuscript fully available?

The [PLOS Data policy](http://www.plosone.org/static/policies.action#sharing) requires authors to make all data underlying the findings described in their manuscript fully available without restriction, with rare exception (please refer to the Data Availability Statement in the manuscript PDF file). The data should be provided as part of the manuscript or its supporting information, or deposited to a public repository. For example, in addition to summary statistics, the data points behind means, medians and variance measures should be available. If there are restrictions on publicly sharing data—e.g. participant privacy or use of data from a third party—those must be specified.

Reviewer #1: Yes

Reviewer #2: No

4. Is the manuscript presented in an intelligible fashion and written in standard English?

PLOS ONE does not copyedit accepted manuscripts, so the language in submitted articles must be clear, correct, and unambiguous. Any typographical or grammatical errors should be corrected at revision, so please note any specific errors here.

Reviewer #1: Yes

Reviewer #2: Yes

5. Review Comments to the Author

Please use the space provided to explain your answers to the questions above. You may also include additional comments for the author, including concerns about dual publication, research ethics, or publication ethics. (Please upload your review as an attachment if it exceeds 20,000 characters)

**Reviewer #1:** The manuscript is technically sound and the conclusions derived are well supported by the available data and statistical analysis performed.
Authors have also performed appropriate statistical analysis for the available data to derive at the results and conclusions from the study.
Authors have also made accessible relevant data to support the findings of the study.
The manuscript was also presented in clear and well communicated English.
The reviewer however will want the authors to address the following

*Replace "this" with "the", in 'this percentage for those selected for analyzing', line 302.

# Authors’ Response

## Thank you very much for reviewing our paper. We have effected the change as per your suggestion. Please see the line 327 (All the line numbers refer to the file named “Revised Manuscript with Track Changes”.).

*By what measure was systematic excluded? , in the statement, 'No remarkable systematic bias was observed in the proportions of women with different characteristics interviewed in the pre- and post lockdown periods.'. Lines 307-309

# Authors’ Response

## Thanks for identifying this confusing sentence. We have corrected the sentence. Please see the lines 332–334.

* Re format Table 2, line 346. The figures in the columns were mal aligned with the rows titled, "Religion, Wealth status, Residence and Media access"

# Authors’ Response

## These corrections have been done. Please see the rows of Religion, Wealth status, Residence and Media access in Table 2. Besides these, we have examined other Tables as well, and have revised the inconsistent alignments and typographic mistakes.

Reviewer #2: Thank you for your effort in this study,:
I think you only assess the effect of sociodemographic characters on the fertility behavior, so I think that must be presented in the title of the research .
This drive us to the question why you didn't assess other factors related to health status or infection with covid?? I think these factors are important and gave role in the fertility motives of the population

# Authors’ Response

## Thank you very much for reviewing our paper and for your valuable comments on enhancing its quality. Our main independent variable in question was whether the respondent was interviewed before or after the lock-down due to the COVID-19 pandemic. Using this classification of interview period, we tried to understand whether the average response to a dependent variable changed after imposing the lockdown. This interview period measured the effect on the behavioural changes of both who had COVID-19 and those who did not have that disease but were at risk of having that. In measuring this effect using multivariate model (logit model), we used the included sociodemographic factors (considered relevant in analysing the behaviour) as control variable in the model, and these control variables were not the primary focus of our study. The analysis of the relationships of the dependent variables with the interview period (whether interviewed before or after lockdown due to the COVID-19 Pandemic) revealed the impact of the spread of the corona virus, i.e. the onset of the COVID-19 pandemic.

## It would be great if we would have been able to include a variable on infection with COVID-19, but there was no variable that could provide information regarding COVID-19 infection. When a pandemic sets on the population of a territory, that affects some people and put others in the risk of having the disease and other kinds of problems which could affect health and financial condition, and social life of both infected and exposed people and others in the recent future. We have explained these possibilities in our manuscript in the lines 70–85 (All the line numbers refer to the file named “Revised Manuscript with Track Changes”.). As a result, a pandemic is expected to affect behaviour of both who are infected and who are at the risk of infection and others in the community. Our main independent variable–Interview period (whether interviewed before or after the COVID-19 lockdown)–measures the impact of pandemic on the behavioural changes in both infected people and those who were at the risk of infection and others in the community.

Please make the limitation of the study and recomendations more clear

# Authors’ Response

We have revised and clarified the limitations and recommendation section. Please see the lines 510–567.

Besides these, we have provided data availability information in the lines 208 – 210 and in the manuscript submission process.

6. PLOS authors have the option to publish the peer review history of their article ([what does this mean?](https://journals.plos.org/plosone/s/editorial-and-peer-review-process#loc-peer-review-history)). If published, this will include your full peer review and any attached files.

If you choose “no”, your identity will remain anonymous but your review may still be made public.


**Do you want your identity to be public for this peer review?** For information about this choice, including consent withdrawal, please see our [Privacy Policy](https://www.plos.org/privacy-policy).

Reviewer #1: No

Reviewer #2: No

[NOTE: If reviewer comments were submitted as an attachment file, they will be attached to this email and accessible via the submission site. Please log into your account, locate the manuscript record, and check for the action link "View Attachments". If this link does not appear, there are no attachment files.]

While revising your submission, please upload your figure files to the Preflight Analysis and Conversion Engine (PACE) digital diagnostic tool, <https://pacev2.apexcovantage.com/>. PACE helps ensure that figures meet PLOS requirements. To use PACE, you must first register as a user. Registration is free. Then, login and navigate to the UPLOAD tab, where you will find detailed instructions on how to use the tool. If you encounter any issues or have any questions when using PACE, please email PLOS at [figures@plos.org](mailto:figures@plos.org). Please note that Supporting Information files do not need this step.

# Authors’ Response

We have revised our figures as per PLOS requirements using PACE.

*In compliance with data protection regulations, you may request that we remove your personal registration details at any time.* [*(Remove my information/details)*](https://www.editorialmanager.com/pone/login.asp?a=r)*. Please contact the publication office if you have any questions.*

Thank you very much for your time and consideration.
