# Supplementary material for: The impact of COVID-19 pandemic on fertility behaviour in Indian states: Evidence from the National Family Health Survey (2019/21)
Source: PLoS One. 2024 Dec 6;19(12):e0314800. doi: 10.1371/journal.pone.0314800 (PMC11623806; doi:10.1371/journal.pone.0314800)
Supplement: Attachment — Submitted filename: Response to Reviewers.docx [file pone.0314800.s009.docx]

PONE-D-23-42397R1

The impact of COVID-19 pandemic on fertility behaviour in Indian states: Evidence from the National Family Health Survey (2019/21)

PLOS ONE

Dear Dr. Mahfuzur,

Thank you for submitting your manuscript to PLOS ONE. After careful consideration, we feel that it has merit but does not fully meet PLOS ONE’s publication criteria as it currently stands. Therefore, we invite you to submit a revised version of the manuscript that addresses the points raised during the review process.

Please submit your revised manuscript by Nov 17 2024 11:59PM. If you will need more time than this to complete your revisions, please reply to this message or contact the journal office at [plosone@plos.org](mailto:plosone@plos.org). When you're ready to submit your revision, log on to <https://www.editorialmanager.com/pone/> and select the 'Submissions Needing Revision' folder to locate your manuscript file.

Please include the following items when submitting your revised manuscript:

- A rebuttal letter that responds to each point raised by the academic editor and reviewer(s). You should upload this letter as a separate file labeled 'Response to Reviewers'.
- A marked-up copy of your manuscript that highlights changes made to the original version. You should upload this as a separate file labeled 'Revised Manuscript with Track Changes'.
- An unmarked version of your revised paper without tracked changes. You should upload this as a separate file labeled 'Manuscript'.

If you would like to make changes to your financial disclosure, please include your updated statement in your cover letter. Guidelines for resubmitting your figure files are available below the reviewer comments at the end of this letter.

If applicable, we recommend that you deposit your laboratory protocols in [protocols.io](http://protocols.io/) to enhance the reproducibility of your results. Protocols.io assigns your protocol its own identifier (DOI) so that it can be cited independently in the future. For instructions see: [https://journals.plos.org/plosone/s/submission-guidelines#loc-laboratory-protocols](https://c05y1x9s.r.us-east-2.awstrack.me/L0/https:%2F%2Fjournals.plos.org%2Fplosone%2Fs%2Fsubmission-guidelines%23loc-laboratory-protocols/1/010f019250f75bcf-04fc2aa9-29d9-42d6-b839-466822961e13-000000/f2UFzv7pYgHcqs-FiHPPuiDo3N8=179). Additionally, PLOS ONE offers an option for publishing peer-reviewed Lab Protocol articles, which describe protocols hosted on [protocols.io](http://protocols.io/). Read more information on sharing protocols at [https://plos.org/protocols?utm_medium=editorial-email&utm_source=authorletters&utm_campaign=protocols](https://c05y1x9s.r.us-east-2.awstrack.me/L0/https:%2F%2Fplos.org%2Fprotocols%3Futm_medium=editorial-email%26utm_source=authorletters%26utm_campaign=protocols/1/010f019250f75bcf-04fc2aa9-29d9-42d6-b839-466822961e13-000000/vi3VY0J984AQCaXr8Eo59m6j-yU=179).

We look forward to receiving your revised manuscript.

Kind regards,

Ashish Wasudeo Khobragade, MD
Academic Editor
PLOS ONE

Journal Requirements:

Please review your reference list to ensure that it is complete and correct. If you have cited papers that have been retracted, please include the rationale for doing so in the manuscript text, or remove these references and replace them with relevant current references. Any changes to the reference list should be mentioned in the rebuttal letter that accompanies your revised manuscript. If you need to cite a retracted article, indicate the article’s retracted status in the References list and also include a citation and full reference for the retraction notice.

# Authors’ Response

The reference list has been checked, which is found complete and correct. We have included following additional references:

26. Gandotra, M. M., Robert D. Retherford, Arvind Pandey, Norman Y. Luther, and Vinod Mishra. 1998. *Fertility in India*. 9. Mumbai, India and Honolulu, Hawaii, U.S.A.: International Institute for Population Sciences and East-West Center Program on Population.

27. Bhat, P. N. Mari, and A. J. Francis Zavier. 2003. “Fertility Decline and Gender Bias in Northern India.” *Demography* 40(4):637–57. doi: 10.2307/1515201.

38. International Institute for Population Sciences (IIPS). National Family Health Survey 2019-20 NFHS-5: Data quality assurance and quality control mechanisms 2019-20 [Internet]. Mumbai, India; 2019 [cited 2024 Oct 18]. Available from: https://www.nfhsiips.in/nfhsuser/assets/pdf/NFHS%20data%20quality%20assurance.pdf

39. Singh SK, Lhungdim H, Shekhar C, Dwivedi LK, Pedgaonkar S, James KS. Innovative field procedures in a large-scale survey to ensure quality of data in pandemic situation: Evidence from NFHS-5, 2019-21. Demogr India. 2022; 51(1):17–39.

48. Kramer S. Religious composition of India. Pew Research Center [Internet]. 2021 [cited 2024 Oct 19]. Available from: https://www.pewresearch.org/wp-content/uploads/sites/20/2021/09/PF_09.21.21_Religious-Composition-of-India-FULL.pdf

51. Aassve A, Le Moglie M, Mencarini L. Trust and fertility in uncertain times. Popul Stud. 2021; 75(1):19–36.

55. Masoudi M, Maasoumi R, Bragazzi NL. Effects of the COVID-19 pandemic on sexual functioning and activity: a systematic review and meta-analysis. BMC Public Health. 2022; 22(1):189.

56. Jacob L, Smith L, Butler L, Barnett Y, Grabovac I, McDermott D, et al. Challenges in the practice of sexual medicine in the time of COVID-19 in the United Kingdom. J Sex Med. 2020; 17(7):1229.

**[Note: HTML markup is below. Please do not edit.]

Reviewers' comments:**

Reviewer's Responses to Questions

**Comments to the Author**

1. If the authors have adequately addressed your comments raised in a previous round of review and you feel that this manuscript is now acceptable for publication, you may indicate that here to bypass the “Comments to the Author” section, enter your conflict of interest statement in the “Confidential to Editor” section, and submit your "Accept" recommendation.

Reviewer #1: All comments have been addressed

Reviewer #2: All comments have been addressed

Reviewer #3: (No Response)

2. Is the manuscript technically sound, and do the data support the conclusions?

The manuscript must describe a technically sound piece of scientific research with data that supports the conclusions. Experiments must have been conducted rigorously, with appropriate controls, replication, and sample sizes. The conclusions must be drawn appropriately based on the data presented.

Reviewer #1: (No Response)

Reviewer #2: No

Reviewer #3: Partly

3. Has the statistical analysis been performed appropriately and rigorously?

Reviewer #1: (No Response)

Reviewer #2: Yes

Reviewer #3: No

4. Have the authors made all data underlying the findings in their manuscript fully available?

The [PLOS Data policy](http://c05y1x9s.r.us-east-2.awstrack.me/L0/http:%2F%2Fwww.plosone.org%2Fstatic%2Fpolicies.action%23sharing/1/010f019250f75bcf-04fc2aa9-29d9-42d6-b839-466822961e13-000000/uLj2qJfa6p0i9BDIT35B0A_kp-o=179) requires authors to make all data underlying the findings described in their manuscript fully available without restriction, with rare exception (please refer to the Data Availability Statement in the manuscript PDF file). The data should be provided as part of the manuscript or its supporting information, or deposited to a public repository. For example, in addition to summary statistics, the data points behind means, medians and variance measures should be available. If there are restrictions on publicly sharing data—e.g. participant privacy or use of data from a third party—those must be specified.

Reviewer #1: (No Response)

Reviewer #2: Yes

Reviewer #3: No

5. Is the manuscript presented in an intelligible fashion and written in standard English?

PLOS ONE does not copyedit accepted manuscripts, so the language in submitted articles must be clear, correct, and unambiguous. Any typographical or grammatical errors should be corrected at revision, so please note any specific errors here.

Reviewer #1: (No Response)

Reviewer #2: Yes

Reviewer #3: No

6. Review Comments to the Author

Please use the space provided to explain your answers to the questions above. You may also include additional comments for the author, including concerns about dual publication, research ethics, or publication ethics. (Please upload your review as an attachment if it exceeds 20,000 characters)

Reviewer #1: (No Response)

Reviewer #2: Thank you for your effort in addressing all my comments.
I hope you to take the comments in consideration in your future researches
I wish you good luck

**Reviewer #3:** This paper addresses a valuable topic and offers some great insights into the impact of COVID-19 on fertility behaviors. That said, there are a few areas that could be improved. Providing more background information would help set the stage for readers from other cultural backgrounds, and improving the overall flow of the argument could make it easier to follow. Also, expanding the statistical analysis section with more details would be helpful for clarity and reproducibility. With these adjustments, the paper could be even stronger.

# Authors’ Response

The authors would like to thank the reviewer for his contribution to improve our paper. We have addressed all the issues showed by the reviewer and respond the questions of the reviewer with due consideration. We have thoroughly revised the paper and tried to improve the clarification beyond the comment of the reviewer (for example ls 269–276). ls means lines and all the line numbers refer to the lines in the file named “Revised Manuscript with Track Changes”

**1.** It would be helpful to give more background on India’s fertility trends for readers who might not be familiar with the country’s unique situation. E.g., explain a bit more about why the mean ideal family size and fertility rate have been dropping over the years?

# Authors’ Response

We have added an explanation on the aforementioned matter in the ls 92–102.

**2.** Make necessary connections/transitions in the introduction section. E.g., “Since the pandemic disrupted these trends, it might be worth expanding on how exactly COVID-19 impacted fertility behaviors.”

# Authors’ Response

This suggestion has been effected in the ls 103–108.

**3.** The terms "direction" and "tempo" of fertility change are important but may benefit from further explanation, particularly for an international audience. A deeper explanation of these concepts in the context of pandemic-related disruptions would add depth to the discussion and broaden the accessibility of the paper for readers unfamiliar with India's demographic landscape.

# Authors’ Response

The terms "direction" and "tempo" of fertility were vague in the context. We have replaced those terms by an appropriate word that we actually meant. Please see the ls 103–106.

**4.** Line 101: “A study showed that fertility intentions in the United Kingdom, …” – add citation of this study.

# Authors’ Response

Citation added. Please see the line 115

**5.** In Socioeconomic profiles section, you provided MPR for all 13 states. Where does these numbers come from? Proper reference is needed to ensure robustness.

# Authors’ Response

We have made the citation clear in the ls 181–187.

**6.** Accurate data is crucial for producing robust results. Figure 3 are very blurry and not readable so I’m not sure whether the following question have been addressed there: What measures have been put in place to ensure that respondents provide truthful information on sensitive topics like their desire for birth within the next two years, current contraceptive use, and recent sexual activity? This information is highly personal.

# Authors’ Response

The figures we provided were very clear, but unfortunately figures got blurred when the complete manuscript file was reproduced by the PLOSONE submission tool. So, we think here the PLOSONE submission tool has scope of work.

NFHS follows ethical protocols at every level of data collection and processing, including informed consent and voluntary participation, ensuring the privacy and confidentiality of data. The field investigators went through rigorous training on survey tools and interview techniques, including gathering sensitive personal information (For example, desire for birth within the next two years, current contraceptive use, and recent sexual activity). NFHS further adheres to the protocol of maintaining a gender-sensitive team composition with a mix of an adequate number of male and female investigators who belong to the native state and have proficiency in the local language(s). Further, a multi-layer monitoring of fieldwork is adopted to strengthen the data quality, including spot checks, back checks, review of field check tables and continuous supportive supervision. Field staff from IIPS conducted spot and back checks of surveyed households in a minimum of 10% of PSUs that were randomly selected by the IIPS central office. The questions used in the NFHSs are integral parts of DHS modules and are asked to women in around 90 countries where DHS surveys are being conducted

(References

1. International Institute for Population Sciences (IIPS). 2019. *National Family Health Survey 2019-20 NFHS-5: Data Quality Assurance and Quality Control Mechanisms 2019-20*. Mumbai, India.
1. Singh, S. K., H. Lhungdim, Chander Shekhar, L. K. Dwivedi, S. Pedgaonkar, and K. S. James. 2022. “Innovative Field Procedures in a Large-Scale Survey to Ensure Quality of Data in Pandemic Situation: Evidence from NFHS-5, 2019-21.” *Demography India* 51(1):17–39.)

We have collated some important information regarding the accuracy of data in the manuscript in the ls 240–252.

**7.** All figures are very blurry and not legible. Higher resolution figures are needed.

# Authors’ Response

The figures we provided were very clear, but unfortunately figures got blurred when the complete manuscript file was reproduced by the PLOSONE submission tool. So, we think here the PLOSONE submission tool has scope of work.

**8.** Line 245: what’s the definition of “clusters” here?

# Authors’ Response

Definition of cluster was provided in the ls 238–239.

**9.** Line 329: “Among the women interviewed in the pre- and post-lockdown periods, more than 80% were Hindu” – elaborate more on how this relates to the analysis. Are Hindus more comfortable to be interviewed compared with others? More context/background information are needed here.

# Authors’ Response

Actually, that section briefly represents the group sizes among which the analysis was performed. Such high percentage of Hindu respondents was because of the fact that around 79% of people in India are Hindu (ref: Kramer, Stephanie. 2021. *Religious Composition of India*. Pew Research Center.). We have put a note on this fact in the ls 367–368.

**10.** The Statistical Analysis section should clearly explain why the two-level mixed effects logit model was used for multivariate analysis. E.g. The data in this study is organized hierarchically: Individuals (level 1) are nested within clusters (level 2) - use two-level mixed effects logit model to address this structure; Capturing Cluster-Level Variation, etc. Current description is not clearly for repeating the analysis.

# Authors’ Response

We have made the description clear in the ls 327–332.

**11.** Tables: provide CI calculation method as table footnotes.

# Authors’ Response

We have provided suggested footnotes. Please see Table 1 and supplementary tables S3, S4, and S5 Tables.

**12.** Why were the 13 states classified based on socioeconomic status? Several conclusions are mentioned in the discussion section. For example, in states with poorer socioeconomic conditions (such as Uttar Pradesh, Jharkhand, and Odisha), contraceptive use significantly declined after the lockdown. It is necessary to further explain the possible reasons for these phenomena, in order to better understand why differences in socioeconomic conditions lead to such disparities.

# Authors’ Response

We added the ls 178–180 to make it clear why we discussed and classified the states and union territories according to socioeconomic status.

We have revised the explanations of different conclusions (that we made) to make those explanations clearer. Our revisions regrading this include ls 477–487, ls 518–523, and ls 530–540.

7. PLOS authors have the option to publish the peer review history of their article ([what does this mean?](https://c05y1x9s.r.us-east-2.awstrack.me/L0/https:%2F%2Fjournals.plos.org%2Fplosone%2Fs%2Feditorial-and-peer-review-process%23loc-peer-review-history/1/010f019250f75bcf-04fc2aa9-29d9-42d6-b839-466822961e13-000000/5vy6MQ4PdDlOMLpTrOHvN-PBfj0=179)). If published, this will include your full peer review and any attached files.


If you choose “no”, your identity will remain anonymous but your review may still be made public.

# Authors’ Response

Authors consent for publishing the peer review history.

**Do you want your identity to be public for this peer review?** For information about this choice, including consent withdrawal, please see our [Privacy Policy](https://c05y1x9s.r.us-east-2.awstrack.me/L0/https:%2F%2Fwww.plos.org%2Fprivacy-policy/1/010f019250f75bcf-04fc2aa9-29d9-42d6-b839-466822961e13-000000/brPz-67xoTwltG1rdMKDJG8boMw=179).

Reviewer #1: No

Reviewer #2: No

Reviewer #3: **Yes:** Yuhang Liu

[NOTE: If reviewer comments were submitted as an attachment file, they will be attached to this email and accessible via the submission site. Please log into your account, locate the manuscript record, and check for the action link "View Attachments". If this link does not appear, there are no attachment files.]

While revising your submission, please upload your figure files to the Preflight Analysis and Conversion Engine (PACE) digital diagnostic tool, [https://pacev2.apexcovantage.com/](https://c05y1x9s.r.us-east-2.awstrack.me/L0/https:%2F%2Fpacev2.apexcovantage.com%2F/1/010f019250f75bcf-04fc2aa9-29d9-42d6-b839-466822961e13-000000/5LFLABrHGkE-MdHrbAvf2k2UMfc=179). PACE helps ensure that figures meet PLOS requirements. To use PACE, you must first register as a user. Registration is free. Then, login and navigate to the UPLOAD tab, where you will find detailed instructions on how to use the tool. If you encounter any issues or have any questions when using PACE, please email PLOS at [figures@plos.org](mailto:figures@plos.org). Please note that Supporting Information files do not need this step.
